# Cx43 and Associated Cell Signaling Pathways Regulate Tunneling Nanotubes in Breast Cancer Cells

**DOI:** 10.3390/cancers12102798

**Published:** 2020-09-29

**Authors:** Alexander Tishchenko, Daniel D. Azorín, Laia Vidal-Brime, María José Muñoz, Pol Jiménez Arenas, Christopher Pearce, Henrique Girao, Santiago Ramón y Cajal, Trond Aasen

**Affiliations:** 1 Patologia Molecular Translacional, Vall d’Hebron Institut de Recerca (VHIR), Vall d’Hebron Hospital Universitari, Vall d’Hebron Barcelona Hospital Campus, Passeig Vall d’Hebron 119-129, 08035 Barcelona, Spain; alexander.tishchenko@ugent.be (A.T.); d.dominguezazorin@dkfz-heidelberg.de (D.D.A.); laia.vidal.b@gmail.com (L.V.-B.); maria.munoz@vhir.org (M.J.M.); pol.jimenezarenas@gmail.com (P.J.A.); chrispearce99@yahoo.co.uk (C.P.); sramon@vhebron.net (S.R.yC.); 2 Biomedical Research (iCBR), Faculty of Medicine, University of Coimbra, Azinhaga de Santa Comba, Celas, 3000-548 Coimbra, Portugal; hmgirao@fmed.uc.pt; 3 Center for Innovative Biomedicine and Biotechnology, University of Coimbra, 3000-548 Coimbra, Portugal; 4 Clinical Academic Centre of Coimbra, CACC, 3000-548 Coimbra, Portugal; 5 Anatomía Patológica, Vall d’Hebron Hospital Universitari, Vall d’Hebron Barcelona Hospital Campus, Passeig Vall d’Hebron 119-129, 08035 Barcelona, Spain; 6 CIBER de Cáncer (CIBERONC), Instituto de Salud Carlos III, Avenida de Monforte de Lemos 3-5, 28029 Madrid, Spain; 7 Universitat Autònoma de Barcelona, 08193 Bellaterra, Spain

**Keywords:** connexin 43, gap junctions, cancer, intercellular communication, breast cancer, cell signaling, tunneling nanotubes, cell–cell communication, tumor microtubes

## Abstract

**Simple Summary:**

The gap junction protein connexin 43 (Cx43) facilitates direct intercellular communication and displays a complex dichotomous role in cancer. Here, we used breast cancer cell lines and CRISPR/Cas9 technology to dissect some of the poorly understood multimodal functions of Cx43 in this setting. Our data show that Cx43 directly modulates the formation of pro-tumorigenic tunneling nanotubes (TNTs), which are cellular protrusions that connect non-adjacent cells to facilitate cellular communication, including the direct cell–cell transfer of organelles. We identify several important cancer signaling pathways that affect TNT formation and show that this effect is strongly influenced by the presence or absence of Cx43. Finally, we demonstrate that Cx43 can affect TNT formation by modulating the cellular secretome. This work provides important insight into the pro-tumorigenic role of Cx43 and its interconnections with TNTs.

**Abstract:**

Connexin 43 (Cx43) forms gap junctions that mediate the direct intercellular diffusion of ions and small molecules between adjacent cells. Cx43 displays both pro- and anti-tumorigenic properties, but the mechanisms underlying these characteristics are not fully understood. Tunneling nanotubes (TNTs) are long and thin membrane projections that connect cells, facilitating the exchange of not only small molecules, but also larger proteins, organelles, bacteria, and viruses. Typically, TNTs exhibit increased formation under conditions of cellular stress and are more prominent in cancer cells, where they are generally thought to be pro-metastatic and to provide growth and survival advantages. Cx43 has been described in TNTs, where it is thought to regulate small molecule diffusion through gap junctions. Here, we developed a high-fidelity CRISPR/Cas9 system to knockout (KO) Cx43. We found that the loss of Cx43 expression was associated with significantly reduced TNT length and number in breast cancer cell lines. Notably, secreted factors present in conditioned medium stimulated TNTs more potently when derived from Cx43-expressing cells than from KO cells. Moreover, TNT formation was significantly induced by the inhibition of several key cancer signaling pathways that both regulate Cx43 and are regulated by Cx43, including RhoA kinase (ROCK), protein kinase A (PKA), focal adhesion kinase (FAK), and p38. Intriguingly, the drug-induced stimulation of TNTs was more potent in Cx43 KO cells than in wild-type (WT) cells. In conclusion, this work describes a novel non-canonical role for Cx43 in regulating TNTs, identifies key cancer signaling pathways that regulate TNTs in this setting, and provides mechanistic insight into a pro-tumorigenic role of Cx43 in cancer.

## 1. Introduction

Twenty-one human connexin proteins form the main structural units of gap junctions, which are intercellular channels that allow the direct diffusion of ions and small molecules between neighboring cells [1]. The critical importance of connexins and gap junctional intercellular communication (GJIC) in normal tissue physiology is evident from the multitude of human diseases associated with connexin mutations and the dysregulation of gap junctions observed in many pathological conditions [2]. More than 50 years of research show that GJIC and connexins are implicated in cancer progression and often act as tumor suppressors [3]. This has led to a significant drive toward the use of connexins as prognostic markers and therapeutic targets in cancer [4]. Although considered tumor suppressors, emerging evidence suggests that connexins can promote tumor growth and survival, particularly in advanced metastatic disease states [3,4,5].

In addition to facilitating GJIC, connexins are thought to possess channel-independent functions. Numerous such functions have been described for connexin 43 (Cx43), the most widely expressed and studied connexin. Notably, many protein–protein interactions in the Cx43 C terminus are thought to regulate important cancer signaling pathways such as c-Src [6]. Interestingly, Cx43 can take up residence in unexpected intracellular compartments, including the inner membrane of mitochondria and the nucleus [7], and Cx43 has been ascribed a functional role in exosomes [8,9]. Furthermore, we and others have shown that Cx43 produces a single mRNA transcript that is capable of synthesizing various N-terminally truncated isoforms through a unique mechanism of direct internal translation initiation [10,11,12]. The major truncated form, named GJA1-20k, corresponds to the C-terminal tail of Cx43 and can interact with numerous proteins and regulate malignant features in cancer cells, including proliferation, migration, and epithelial-to-mesenchymal transition (EMT) [6]. Notably, GJA1-20k modulates the motility and biogenesis of mitochondria [13,14] and regulates actin dynamics [15]. GJA1-20k has also been described in exosomes [16] and, surprisingly, in the nucleus, where it may act as a transcriptional regulator of N-cadherin during EMT [17]. These emerging non-canonical functions of Cx43 are providing new biological insight into cancer, as well as therapeutic opportunities [4,18]. However, dissection of the channel-dependent and -independent functions driving both the anti- and pro-tumorigenic pathways remains a major challenge.

Tunneling nanotubes (TNTs), first described in 2004 [19], are thin, actin-based, membrane bridges enclosed in a lipid bilayer that connect cells over distances of up to several cell diameters [20,21]. A wide variety of TNT-mediated cargo transport has been described, including that of ions [22], miRNAs [23], cytosolic proteins [24], and prions [25,26,27], pathogens, such as viruses [28,29] and bacteria [30], and organelles, such as the endoplasmic reticulum [31], mitochondria [32], Golgi vesicles [31], endosomes [33], and lysosomes [33]. Due to the heterogeneous nature of these transient structures, the terms “thin” and “thick” have been used to differentiate between TNTs with smaller (<0.7 µm) and larger (>0.7 µm) diameters, respectively [33,34]. “Thin” TNTs contain mainly F-actin, whereas “thick” TNTs tend to contain both actin and microtubules. TNTs may originate when two joined cells move apart from each other and remain connected by a thin membranous thread [35] or when filopodia-like protrusions from one cell dock with a nearby cell [19]. These models are not mutually exclusive. The underlying molecular mechanisms are not fully understood and likely depend on the specific cell type and its environment. Indeed, it appears that several types of TNT-like structures exist, including what are known as tumor microtubes, which are longer and seemingly more stable [36]. A better understanding of the molecular mechanisms underlying the formation and functions of TNTs is necessary to fully understand their physiological and pathological roles.

Cellular stress, such as hypoxia or a low-serum hyperglycemic medium, seems to stimulate TNT formation. In particular, TNTs have been described in several cancer cell lines, as well as between cancer cells and stromal cells (reviewed in [37,38]). TNTs have also been found in primary human tumors [39,40,41]. It is becoming clear that TNTs facilitate a pathway of intercellular conduits in tumors [42] and may promote numerous malignant features such as invasion, growth, chemo- and radiotherapy resistance, and surgery-induced tumor growth [43,44,45].

Connexins localize to TNTs and regulate intercellular communication [44,46,47]. Wang et al. [46] identified TNT-mediated voltage-sensitive electrical coupling that was inhibited by the gap junction inhibitor meclofenamate. Cx43 immunoreactivity was observed at one end of the TNTs. Lock et al. [48] showed that TNTs between HeLa cells created cytosolic continuity allowing inositol triphosphate-mediated calcium signaling, but only if a pair of cells were both expressing Cx43. More recently, human immunodeficiency virus (HIV) infection was shown to stimulate TNT formation and connect human macrophages. These TNTs contained Cx43 at the tip, and treatment with the GJIC inhibitors 18-alpha-glycyrrhetinic acid or octanol prevented TNT-mediated cell–cell communication and viral cell–cell spread [49]. Although these inhibitors are nonspecific and affect several other targets and channel proteins, the findings suggest that Cx43 channels may be required for the functional establishment of TNTs that then allow the transfer of larger structures (including HIV) that, due to size constraints, do not pass through gap junctions. Notably, recent work suggests that Cx43 regulates TNT formation in normal human trabecular meshwork cells [50]. Yao et al. showed that Cx43 mediates TNT formation and mitochondrial transfer from human-induced pluripotent stem cell-derived mesenchymal stem cells and mouse epithelial cells [51]. Mechanistically, Fykerud et al. [52] showed that Cx43 remodeling was driving the formation of actin-based plasma membrane bridges (termed “mitotic nanotubes”) with adjacent cells during mitosis, facilitating vesicle transfer between cells. In vivo, glioma cells have been described to form a network of TNT-like structures called microtubes that contribute to tumor invasion in the brain [40]. Cx43-containing gap junctions within this network are thought to turn the tumor into a syncytium of interconnected cells that is highly resistant to radiation therapy, presumably by distributing calcium between cells to prevent apoptosis upon the radiation-induced release of intracellular calcium [40]. Brain tumors appear to employ a similar system of microtubes or TNTs to establish connections with neurons to “feed off the brain” [53]. Similarly, breast cancer cells that metastasize to the brain benefit from the formation of pseudo-tripartite synapses between cancer cells and glutamatergic neurons to promote metastatic growth [54,55]. Indeed, several studies have described the presence of TNTs in breast cancer cells and between breast cancer cells and macrophages, facilitating drug resistance, invasion, and metastasis [56,57,58,59]. A large body of data suggests that Cx43 is intricately linked to breast cancer development and may act as a tumor suppressor, particularly at early stages (see reviews [60,61,62,63,64]). However, increasing evidence suggests that some breast cancers overexpress Cx43, which may facilitate certain malignant and metastatic capabilities. Most notably, the expression of Cx43 was shown to create a syncytium between breast cancer cells and astrocytes, forming a cyclic guanosine monophosphate–adenosine monophosphate (cGAMP)-driven paracrine feedback loop that supports tumor growth and chemoresistance [65]. Consequently, the GJIC inhibitor meclofenamate is under clinical trial for breast cancer patients with brain metastasis (https://clinicaltrials.gov/ct2/show/NCT02429570).

Considering the emerging evidence linking Cx43 and TNTs, we sought to further explore this interplay in breast cancer cells by knocking out Cx43 expression using CRISPR-Cas9 technology. We show that Cx43 significantly regulates the formation and length of TNTs in breast cancer cells. Notably, TNT stimulation was more potent with conditioned medium derived from Cx43-expressing cells than with that from Cx43 knockout (KO) cells. Furthermore, several pathways that are regulated by Cx43 were identified to induce TNTs. Some of these pathways are novel TNT modulators. These pathways also regulate Cx43 function, and thus the direct association needs to be explored further. Specifically, Cx43 KO cells were more sensitive to drug-induced TNT formation than wild type (WT) cells. Further elucidation of the role of Cx43 in this setting is an exciting cutting-edge challenge that can provide mechanistic insight into the pro-metastatic features of Cx43 and the regulation of TNTs.

## 2. Results

### 2.1. GJA1 Knockout Reduces TNT Number and Length in BT549 Breast Cancer Cells

To achieve the full and long-term elimination of Cx43 expression from cells, we developed an all-in-one transient CRISPR/Cas9 expression plasmid that targets *GJA1* (encoding Cx43) and truncated forms of the protein such as GJA1-20k. The well-established PX458 plasmid allows the expression of CRISPR targeting sequences together with an EGFP-linked form of Cas9 to control transfection and fluorescence-activated cell sorting (FACS) selection [66]. To minimize putative off-target cleavage effects, we replaced the standard Cas9 with the VP12 high-fidelity Cas9 version [67]. Specific sequences targeting the coding exon of *GJA1* were introduced. We transfected various cell lines and used FACS to isolate single cells transiently expressing Cas9-EGFP. Clones were screened by western blot to detect loss of Cx43 expression. This tool has allowed us to generate a wide variety of cancer cell lines lacking Cx43 expression to evaluate various phenotypic changes related to malignancy.

**Figure 1:**
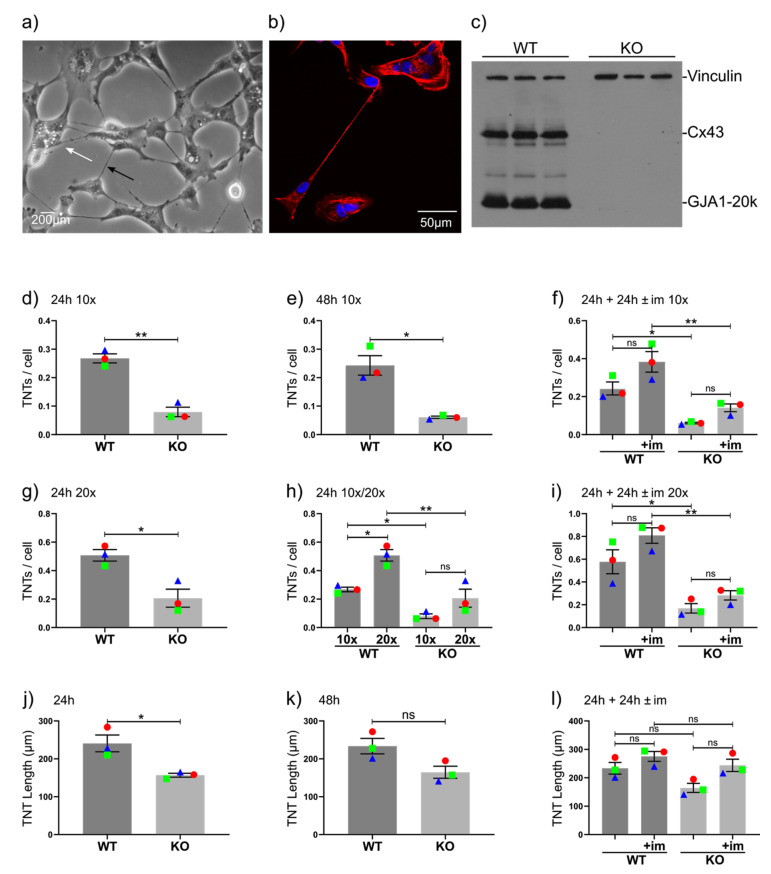
Tunneling nanotubes (TNTs) are regulated by connexin 43 (Cx43) in BT-549 cells. (**a**) Morphological examples of TNTs in BT549 cells (20×). The black arrow denotes a typical “thick” TNT, whereas the white arrow denotes a typical “thin” TNT that is often only visible at higher magnifications. (**b**) Confocal image of BT-549 cells stained with F-actin and the nuclear stain 4′,6-diamidino-2-phenylindole (DAPI) demonstrating a long, thin, F-actin–positive TNT. (**c**) Western blot with independently isolated samples of three wild type (WT) and three CRISPR-Cas9 Cx43 knockout (KO) BT549 cells showing the presence or absence (respectively) of both Cx43 (43 kDa) and the truncated GJA1-20k form (20 kDa), as well as some other weakly expressed truncated forms of Cx43. The blot was co-incubated with a vinculin antibody (124 kDa) as an internal loading control. (**d**,**e**) Average numbers of TNTs per cell in WT and Cx43 KO BT549 cells at 10× (counting thick TNTs) (**d**) 24 h after seeding, (**e**) 48 h after seeding with cells, and (**f**) 24 h after seeding followed by 24 h refreshed with the same medium or in a low-glucose, low-serum inductive medium (im+) to stimulate TNT formation. (**g**) Average numbers of TNTs per cell in WT and Cx43-KO BT549 cells at 20× (counting thick and thin TNTs) 24 h after seeding. (**h**) Comparison of the numbers of TNTs counted at 10× and 20×. (**i**) Average numbers of TNTs at 48 h with or without TNT-inductive medium (im+) for the last 24 h. (**j**–**l**) Average lengths of TNTs at (**j**) 24 and (**k**) 48 h and (**l**) 48 h with or without treatment with TNT-inductive medium (im+) for the last 24 h. The means of individual experiments (calculating the average number of TNTs per cell [TNTs/cell] in *n* = 5 images) are highlighted and linked by color and shape to facilitate the tracking of trends between individual experiments. Error bars denote the mean and SEM of three independent biological experiments. Significance was tested with two-way Student’s *t*-test for single comparisons and one-way ANOVA with Tukey’s post-hoc analysis for multiple comparisons (* *p* < 0.05, ** *p* < 0.01, ns = not significant).

We noticed that the triple-negative breast cancer cell line BT549 tends to display an extensive TNT network (Figure 1a) that stains for F-actin (Figure 1b). Time-lapse microscopy clearly highlighted this network and showed that the formation of these TNTs may occur through the protrusion of filopodia-like structures that connect to a distant cell and/or through two adjacent cells migrating apart from each other (Supplementary Video S1). We previously showed that this cell line expresses very high levels of full-length Cx43, as well as the 20-kDa truncated form GJA1-20k [10]. We efficiently eliminated *GJA1* expression from this cell line using our CRISPR-Cas9 plasmid (Figure 1c). This led to a marked but not complete loss of GJIC, suggesting that other connexin channels may operate in this cell line (Figure S1). Morphologically, we observed a noticeable reduction in the number of TNTs in knockout (KO) cells compared with WT cells. Detailed quantification demonstrated that the loss of Cx43 expression significantly reduced the average numbers of TNTs, both 24 (Figure 1d) and 48 (Figure 1e) h after seeding.

Next, we decided to deliberately stress cells by culturing them in low-serum hyperglycemic media, which was a condition previously shown to stimulate TNT formation [39]. Using such TNT-inductive media (im+), the number of TNTs noticeably but nonsignificantly increased (Figure 1f). The difference between WT and KO cells was significant under im+ conditions (Figure 1f), altogether suggesting that the response to this specific condition of cellular stress is similar in WT and KO cells. In addition to having noticeably thicker TNTs, BT549 cells also display some very thin TNTs at 20× magnification or higher (see white arrow, Figure 1a). After the inclusion of these “thin” TNTs via the analysis of 20× images, the difference between WT and KO cells remained similar and significant (Figure 1g), together with a significant (and expected) increase in the number of TNTs per cell (Figure 1h). Similar to our 10× analysis, our 20× analysis suggested that low-serum hyperglycemic conditions stimulated TNT formation, albeit nonsignificantly (Figure 1i). The results of the detailed 20× analysis seemed to strongly correspond with our 10× analysis in terms of differences between cells and were not repeated for subsequent analyses.

Since there was a significant decrease in the number of TNTs in KO cells, we also compared lengths. We found a significant reduction in TNT length in the absence of Cx43 (Figure 1j). This was noticeable but not significant at 48 h, possibly because cells are at a higher density at this stage (Figure 1k). TNT-inductive medium (im+) also appeared to increase the length of TNTs in both WT and KO cells (Figure 1l).

### 2.2. GJA1 Knockout Reduces the Number of TNTs in Hs578t Breast Cancer Cells

**Figure 2:**
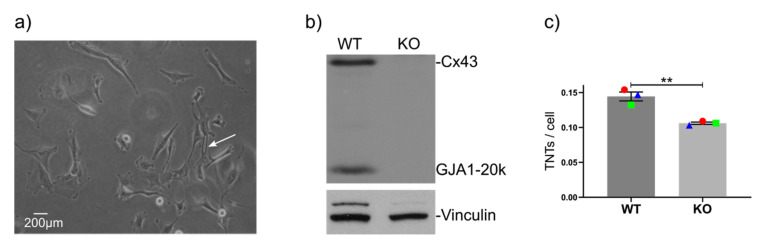
TNTs are regulated by Cx43 in HS578t cells. (**a**) Morphological examples of TNTs in HS578t cells (20×). The white arrow denotes a typical TNT. (**b**) Western blot of WT and CRISPR-Cas9 Cx43KO Hs578t cells showing the presence or absence of Cx43 (43 kDa) and the truncated GJA1-20k form (20 kDa). Vinculin (124 kDa) was used as a loading control. (**c**) Average numbers of TNTs per cell in WT and Cx43-KO Hs578t cells at 10× (counting thick TNTs) 24 h after seeding. The means of individual experiments (calculating the average number of TNTs per cell [TNTs/cell] in *n* = 5 images) are highlighted and linked by color and shape to facilitate the tracking of trends between individual experiments. Error bars denote the mean and SEM of three independent biological experiments. Significance was tested using the two-way Student’s *t*-test (** *p* < 0.01, ns = not significant).

To confirm that the Cx43-mediated regulation of TNT formation is a more general phenomenon and not unique to the BT549 breast cancer cell line, we decided to analyze a second triple-negative breast cancer cell line that expresses high levels of Cx43. We chose to analyze the Hs578t cell line because this Cx43-expressing cell line displayed a considerable amount of TNTs under basal conditions (Figure 2a). Moreover, small interfering RNA-mediated silencing of Cx43 has been shown to affect malignant features in this cell line [68]. We applied CRISPR/Cas9 to knockout Cx43 (and GJA1-20k) expression (Figure 2b). This led to a significant reduction in the number of TNTs (Figure 2c). However, WT Hs578t cells exhibited markedly fewer TNTs compared with BT549 cells (approximately two-fold, Figure 1d vs. Figure 2c). Other breast cancer cell lines do not have an appreciable amount of TNTs (see below). Considering that Hs578t cells display an “intermediate” level of TNTs, we considered this cell line an ideal model to test for any positive or negative effects on TNT formation caused by the targeting of specific pathways known to be regulated by Cx43.

### 2.3. Signaling Pathways Linked to Cx43 Differentially Affect TNT Formation in Cx43 WT and KO Cells

**Figure 3:**
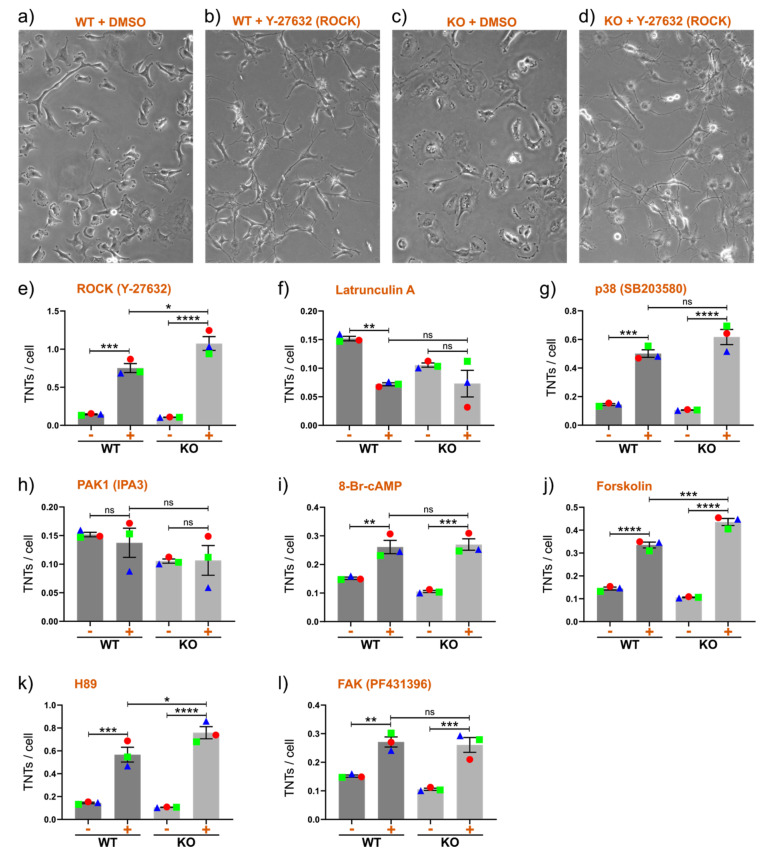
Drug-induced TNT formation in Hs578t cells upon overnight (16-h) stimulation. (**a**–**d**) Examples of morphology and TNTs in WT and KO Hs578t cells stimulated overnight with DMSO control or the RhoA kinase (ROCK) inhibitor Y-27632, which significantly increased the number of TNTs. (**e**–**l**) Quantification of the average numbers of TNTs in WT and KO Hs578t cells in DMSO control (−) and drug-stimulated (+) cells, as specified in each graph. The means of individual experiments (calculating the average number of TNTs per cell [TNTs/cell] in *n* = 5 images) are highlighted and linked by color and shape to facilitate the tracking of trends between individual experiments. Error bars denote the mean and SEM of three independent biological experiments. Significance was tested using one-way ANOVA with Tukey’s post-hoc analysis for multiple comparisons (* *p* < 0.05, ** *p* < 0.01, *** *p* < 0.001, **** *p* < 0.0001, ns = not significant).

TNT formation is closely linked to the cytoskeletal network, with disruption of F-actin polymerization reducing TNT formation [69]. Rho-GTPases regulate actin polymerization, such as during the formation of TNTs [70]. RhoA signaling, including Rho-associated protein kinase (ROCK), is regulated by Cx43 in breast epithelial cells [71], which is an observation supported by us in Hs578t cells (Figure S2). RhoA signaling can also affect Cx43 expression levels and channel activity [72,73,74]. Thus, we decided to inhibit actin stress fiber formation with the ROCK inhibitor Y-27632. In this case, we observed a highly significant increase in the number of TNTs (as illustrated in Figure 3a–d and summarized in Figure 3e). This is consistent with results published for other cell types [69]. Curiously, this effect was even stronger in KO cells (mean increase of 0.97 TNTs/cell vs. a mean increase of 0.61 TNTs/cell in WT cells). This observation reinforces the idea that the effect of Y-27632 is exerted on RhoA signaling and not on any Cx43-related mechanisms. In support of a RhoA-associated mechanism, we observed a trend toward a reduced F-/G-actin ratio in KO cells compared with WT cells (Figure S2). As a control for the critical role of F-actin in TNT formation, we applied the actin polymerization inhibitor latrunculin A, which prevents the assembly of F-actin into supramolecular structures. As expected, this reduced the number of TNTs in Hs578t cells, especially in WT cells, which initially contained more TNTs (Figure 3f).

RhoA-ROCK activates p38 [75], another signaling pathway that is regulated by Cx43 [76] and that regulates Cx43 [6]. We blocked p38 kinase activity using the well-known specific inhibitor SB-203580, which led to a significant increase in the number of TNTs (Figure 3g). Again, an even higher increase was observed in KO cells than in WT cells. This is the first report directly showing that the inhibition of p38 activity can be a potent inducer of TNTs. It is worth noting that Cx43 (or the truncated C-terminal tail of Cx43) activates p38 to induce the formation of filopodia through upstream interaction with and activation of p21-activated protein kinase 1 (PAK1) [76]. The inhibition of both p38 and PAK1 reduces filopodia formation [76]. We also tested the PAK1 inhibitor IPA3 but did not observe any significant changes in WT or KO cells (Figure 3h). However, IPA3 does not efficiently inhibit PAK2, which is a kinase that affects RhoA signaling [77]. The fact that both Cx43 and p38 signaling affect TNT (this study) and filopodia [76] formation in an opposite manner indicates that subtle mechanistic differences are important and is in accordance with reports that the formation of TNTs and filopodia are driven by distinct opposing actions of the actin regulatory complexes [78].

Another signaling pathway that regulates the actin cytoskeleton and F-actin dynamics is the cyclic AMP-dependent protein kinase (cAMP/PKA) pathway [79,80]. PKA also potently regulates Cx43 gap junctions [81], which are the same gap junctions that are a major conduit for cAMP transfer between cells. The activation of cAMP/PKA signaling, using either forskolin or the cAMP analog 8-Br-cAMP, can restore Cx43 gap junctional activity [82]. Notably, activation of PKA by forskolin or 8-Br-cAMP inhibits downstream RhoA-ROCK signaling [83]. In Hs578t cells, treatment with both 8-Br-cAMP (Figure 3i) and forskolin (Figure 3j) significantly induced TNT formation in WT cells, and even more so in KO cells (forskolin reached high significance in a comparison between stimulated WT and KO cells). We also inhibited PKA activity using the drug H89. Surprisingly, this also potently stimulated TNT formation (Figure 3k). However, H89 also specifically inhibits downstream RhoA-ROCK signaling, and H89 might not be suitable as an antagonist of PKA in systems in which ROCK signaling is important [84].

The extracellular signal-regulated kinase (ERK)/RhoA/focal adhesion kinase (FAK) network also regulates the cytoskeletal network. FAK interacts with c-Src to mediate many downstream signaling pathways, including RhoA signaling [85]. Indeed, β1 integrin-mediated modulation of a FAK–RhoA–actomyosin signaling axis regulates membrane protrusions [86]. Notably, Cx43 is phosphorylated by Src and, conversely, a region of the Cx43 C terminus also regulates c-Src activity and Src/FAK signaling [87]. Therefore, we treated cells with the FAK inhibitor PF 431396. This inhibitor significantly increased TNT numbers in both WT and KO cells (Figure 3l). We also tested a short inhibitory peptide known to inhibit FAK/Src activity [87] but did not observe any major difference (Figure S3).

### 2.4. Drug-Induced Stimulation of TNTs in the Presence or Absence of Cx43 in Cell Lines Expressing Very High or Very Low Numbers of TNTs under Normal Conditions

**Figure 4:**
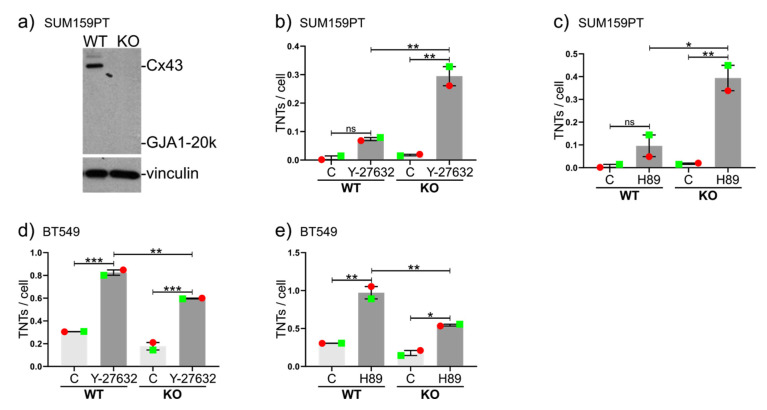
Drug-induced TNT formation in SUM159PT and BT549 cells. (**a**) Western blot of WT and CRISPR-Cas9 Cx43 KO cells showing the presence or absence of Cx43 (43 kDa) in SUM159PT cells. Vinculin (124 kDa) was used as a loading control. (**b**,**c**) Average numbers of TNTs per cell in WT and Cx43 KO SUM159PT cells with or without the inhibitors Y-27632 (**b**) and H89 (**c**). (**d**,**e**) Average numbers of TNTs per cell in WT and Cx43-KO BT549 cells with or without the inhibitors Y-27632 (**d**) and H89 (**e**). The means of individual experiments (calculating the average number of TNTs per cell [TNTs/cell] in *n* = 5 images) are highlighted and linked by color and shape to facilitate the tracking of trends between individual experiments. Error bars denote the mean and SEM of two independent biological experiments. Significance was tested using one-way ANOVA with Tukey’s post-hoc analysis for multiple comparisons (* *p* < 0.05, ** *p* < 0.01, *** *p* < 0.001, ns = not significant).

Since Y-276332 (ROCK inhibitor) and H89 were exceptionally potent at stimulating TNT formation, we decided to test these agents in the breast cancer cell line SUM159PT, which displays very few TNTs under basal conditions, despite expressing some Cx43 (but low levels of GJA1-20k). We also generated a Cx43 KO version of this cell line (Figure 4a). Both Y-276332 (Figure 4b) and H89 (Figure 4c) stimulated the de novo formation of TNTs in both WT and KO cells, although the results were only significant in KO cells. Indeed, TNT induction was significantly more potent in KO cells (16.8-fold and 4.3-fold more potent stimulation in KO cells than in WT for ROCK and H89, respectively). We also confirmed that these inhibitors potently stimulated TNT formation in the BT549 cell line, which already expresses a very high number of TNTs (Figure 4d,e). In this instance, overall TNT induction was slightly less potent in KO cells than in WT cells (although the ratio increase was still higher). A complex interplay between Cx43-related pathways is involved in TNT formation.

### 2.5. Further Insight into Cx43 Pathways Stimulating TNT Formation

Our results show that Cx43 stimulates TNT formation in breast cancer cells. Furthermore, the inhibition of several pathways linked to Cx43, such as p38 and ROCK, appears to stimulate TNT formation more effectively in KO cells. We adopted several experimental approaches to obtain additional insight into the molecular pathways through which Cx43 may affect TNTs.

#### 2.5.1. The role of GJIC

**Figure 5:**
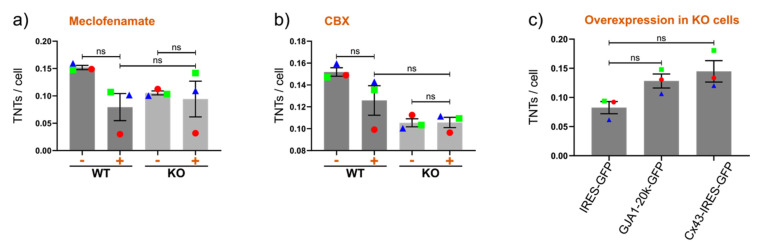
Stimulation of TNT formation through gap junctional intercellular communication (GJIC) and GJA1-20k. (**a**) Inhibition of GJIC by meclofenamate. (**b**) Inhibition of GJIC by carbenoxolone (CBX). (**c**) Overexpression of Cx43 and GJA1-20k in Hs579t Cx43 KO cells. The means of individual experiments (calculating the average number of TNTs per cell [TNTs/cell] in *n* = 5 images) are highlighted and linked by color and shape to facilitate the tracking of trends between individual experiments. Error bars denote the mean and SEM of three or more independent biological experiments as indicated. Significance was tested using one-way ANOVA with Tukey’s post-hoc analysis for multiple comparisons (ns = not significant).

GJIC inhibitors such as meclofenamate and 18-alpha-glycyrrhetinic acid affect TNT-mediated cell coupling without affecting TNT formation because their activity is reversible upon drug washout [46,49]. We tested the GJIC inhibitor meclofenamate in Hs578t cells because these cells mainly express Cx43 and because KO cells are almost devoid of GJIC (Figure S4). Meclofenamate specifically reduced the levels of TNTs in WT cells and not in KO cells, albeit nonsignificantly (Figure 5a). However, flow cytometry analysis of the parachute dye-transfer assay suggested a very modest inhibition of GJIC (Figure S4). Another GJIC inhibitor, carbenoxolone (CBX), a glycyrrhetinic acid derivative (that notably also affects Cx43 levels and inhibits pannexin1 channels), also seemed to specifically reduce TNT levels in WT cells (Figure 5b). Cx43 KO cell lines provides an important tool in this setting to compare GJIC-specific and -unspecific effects (Figure 1c, Figure 2b, Figure 4c and Figure S5). However, due to the nonspecific and potentially toxic effects of these inhibitors and considering the sensitivity of TNTs to injury, for example, additional experiments are required to conclude whether GJIC directly affects TNT formation or only TNT function. Toward this, a preliminary peptide screen indicated that the channel- and hemichannel-inhibiting Cx43 mimetic peptide Gap27 does not seem to have a major effect on TNT levels (Figure S3).

#### 2.5.2. Channel-Independent Effects

On the other hand, regulation of the cytoskeleton (critical for TNT formation) is thought to be mediated by non-junctional features of the Cx43 C terminus [6,88]. For example, the overexpression of the Cx43 C terminus regulates filopodia formation [76], and the independently translated Cx43 C-terminal protein GJA1-20k regulates actin dynamics [15]. Therefore, we overexpressed either GJA1-20k or Cx43 in Hs578t Cx43KO cells and quantified the levels of TNTs. Increased TNT formation was observed (Figure 5c), but the effect was relatively weak and did not reach statistical significance, even in control Cx43-overexpressing cells. Additional approaches, including overexpression in the absence of EGFP, are needed to draw firmer conclusions.

#### 2.5.3. Induction of TNTs by Conditioned Media

**Figure 6:**
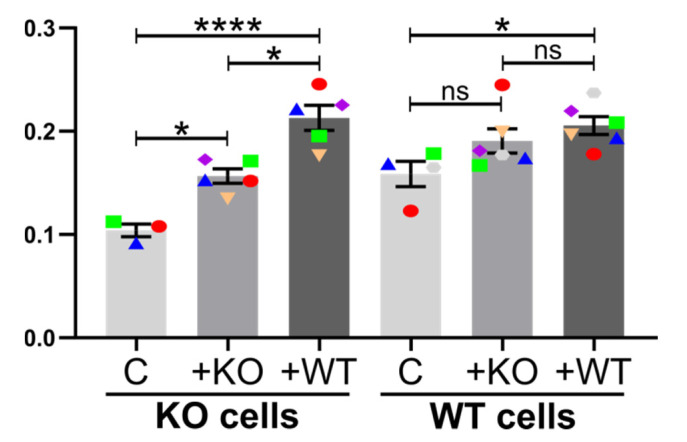
Stimulation of TNT formation by conditioned medium in Hs578t cells. Average numbers of TNTs per cell in WT and Cx43 KO Hs578t cells. C = control conditions. +KO = stimulation with conditioned medium derived from Hs578t Cx43 KO cells. +WT = stimulation with conditioned medium derived from Hs578t WT cells expressing Cx43. The means of individual experiments (calculating the average number of TNTs per cell (TNTs/cell) in *n* = 5 images) are highlighted and linked by color and shape to facilitate the tracking of trends between individual experiments. Error bars denote the mean and SEM of three or more independent biological experiments, as indicated. Significance was tested using one-way ANOVA with Tukey’s post-hoc analysis for multiple comparisons (* *p* < 0.05, **** *p* < 0.0001, ns = not significant).

Previous work has shown that conditioned medium from macrophages stimulates TNT formation in breast cancer cells [57,59]. Cx43 significantly alters the secretome and affects features such as the actin cytoskeleton [89]. Therefore, we tested whether Cx43 affected the ability of conditioned medium to induce TNTs. As seen in Figure 6, conditioned medium stimulated TNT formation. Notably, medium from WT cells was significantly more potent than medium from KO cells, suggesting that the presence of Cx43 at least partly stimulates TNT formation by modifying the secretome. Since Cx43 is present in numerous cell types, it will be of particular interest to elucidate the mechanism of Cx43-driven secretome-mediated TNT stimulation.

## 3. Discussion

In 2004, Rustom et al. [19] published their seminal article describing nanotubular structures termed TNTs that facilitated the selective transfer of membrane vesicles and organelles but seemingly impeded the flow of small molecules such as the gap junction-permeable dye calcein. However, they focused on gap junction-deficient PC12 cells [90], and the same authors and others have since described the presence of gap junction proteins, including Cx43, in TNTs that facilitate electrical and dye coupling between cells [46,48,49,91]. Notwithstanding, Cx43 may have multiple functions in TNTs. Here, we show for the first time that Cx43 contributes to the formation of TNTs in breast cancer cells. This is in agreement with work showing that Cx43 mediates TNT formation and mitochondrial transfer from human-induced pluripotent stem cell-derived mesenchymal stem cells and mouse epithelial cells [51]. Cx43 has also been shown to regulate the amount of TNTs in normal human trabecular meshwork cells [50]. The fact that Cx43 seems to regulate TNTs in cancer cells is particularly interesting given the complex pro- and anti-tumorigenic roles of Cx43 in cancer [3]. Strong evidence for such cooperation was shown by Osswald et al. [40], who demonstrated the presence of Cx43 in TNT-like structures termed tumor microtubes between glioma cells and astrocytes that facilitated tumor growth and therapy resistance. Notably, in recent years, Cx43 has been shown to facilitate metastasis, such as breast and lung carcinoma to the brain [65], and the role of pro-tumorigenic TNTs in these settings needs further exploration.

**Figure 7:**
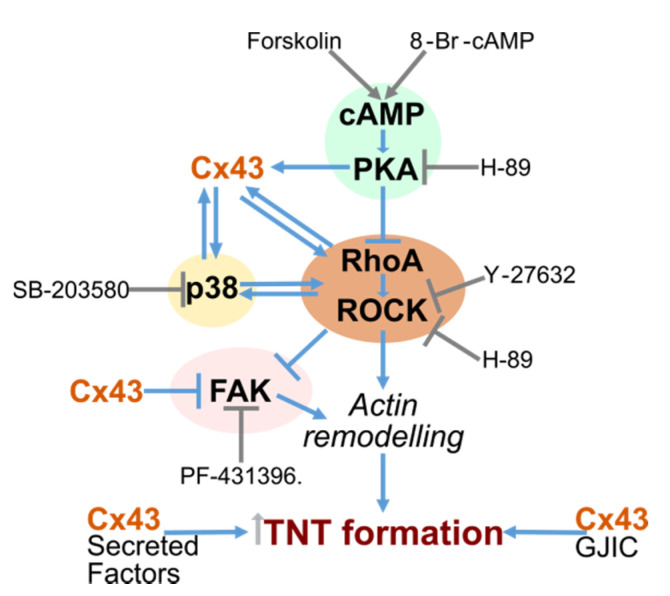
Cell signaling pathways and links to Cx43 and TNT formation.

The formation of TNTs is not fully understood, and the molecular mechanism implicating Cx43 in this process is unknown. Using Cx43 WT and KO cells, we explored various signaling pathways that are known to both regulate Cx43 and be themselves regulated by Cx43. We identified several pathways that significantly affect TNT levels, notably PKA/cAMP, p38, ROCK, and FAK. An intricate inter-relationship between these signaling pathways and Cx43 appears to exist (Figure 7), although the main driving force for TNT formation in this setting remains to be defined.

The three canonical Rho-GTPases—RhoA, Rac1, and Cdc42—are key regulators of different cytoskeletal and adhesion dynamics in cells [92]. Critically, and consistent with our findings, RhoA–ROCK signaling regulates TNTs. For example, others have used the same ROCK inhibitor that we used (Y-27632) to demonstrate increased TNT formation and vesicle transfer via TNTs [69,93,94]. Mechanistically, it has been suggested that this inhibitor may lead to the disassembly of F-actin stress fibers, thereby enriching the cellular pool of G-actin available for TNT formation [69]. RhoA surfaces as a candidate that induces TNTs in this setting, based on multiple reports, including ours, showing that ROCK inhibition stimulates TNTs, whereas the formation of TNT-like protrusions seems to be significantly reduced with the inhibition of either Cdc42 or Rac1 [70]. The fact that the activation of cAMP also stimulated TNTs was not surprising, considering that cAMP, through PKA, phosphorylates and inhibits RhoA membrane translocation and ROCK signaling [95].

A bidirectional relationship exists between Cx43 and the RhoA pathway. For example, RhoA regulates the permeability of Cx43 channels in myocytes [73]. The inhibition of ROCK increases the number of Cx43 gap junctions and GJIC in corneal epithelial cells [96]. It has been suggested that GJIC is reduced via RhoA-mediated Cx43 phosphorylation and Cx43/ZO-1/drebrin complex instability-mediated Cx43 degradation [97]. Curiously, in prion-infected neurons, RhoA seems to interact with Cx43, with RhoA/ROCK inhibition blocking Cx43 hemichannel activity [98]. In agreement with this hypothesis, previous work strongly suggested that RhoA activation is a key player in thrombin-induced inhibition of Cx43 hemichannel activity [74]. Increased F-actin induced by RhoA/ROCK signaling promotes the association between ZO-1 and Cx43, leading to endocytosis and reduced Cx43 expression [72]. However, the potent stimulation of TNTs in Cx43 KO cells suggests that the regulation of TNTs by RhoA/ROCK is more direct and does not occur via Cx43 as a downstream target. Indeed, Cx43 regulates RhoA/ROCK activity. For example, the upregulation of Cx43 expression can reduce NF-κB p65 nuclear translocation induced by RhoA/ROCK signaling [72]. Cx43 knockdown also affects fibroblast cytoskeletal dynamics after scratch-wounding, with cells exhibiting longer lamellipodial protrusions lacking the F-actin belt and activation of Rac-1 and RhoA GTPases [99]. A recent study directly linked Cx43 and the RhoA/ROCK pathway, thus providing an insight into the possible role of Cx43 in TNT formation. Fostok et al. [71] found that Cx43 silencing is associated with ERK1/2 activation and upregulated the expression of all three main Rho-GTPases (RhoA, CDC42, and Rac1) in a non-malignant breast epithelial cell line. Our observation of increased RhoA in Hs578t cells is in line with this observation.

The stimulation of TNTs upon ROCK inhibition was even more potent in Cx43 KO cells. This was a surprising observation that also was seen upon the inhibition of p38. These pathways are closely linked. RhoA–ROCK activates p38 [75] and activation of RhoA induces p38-dependent pseudopodial protrusions [100]. We used multiple breast cancer cell lines to show for the first time that p38 is a direct regulator of TNT formation. However, indirect targeting of p38, by blocking its upstream activator PAK1 with the drug IPA-3, inhibits TNT formation in a neuroblastoma cell line [101]. Moreover, the truncated form of Cx43 stimulates filopodia formation by activating p38 through phosphorylation of the upstream target PAK1, with filopodia formation significantly reduced via IPA-3 inhibition of PAK1 activity [76]. However, the formation of filopodia and TNTs is stimulated through opposing pathways of the actin regulatory network, and the filopodia-promoting CDC42/IRSp53/VASP network negatively regulates TNT formation [78]. Our results are in agreement with this observation, with Cx43 KO cells forming significantly more TNTs in response to p38 inhibition compared with Cx43-expressing cells, which explains why p38 inhibition may reduce filopodia formation but induce TNTs in breast cancer cell lines. The role of PAK1 in this setting remains unclear, because IPA-3 did not significantly affect TNT levels.

p38 induces stress fiber formation, which can be blocked by p38 inhibition [102,103]. RhoA and FAK are also critical regulators of actin stress fibers [104]. Moreover, the improper alignment of actin stress fibers is observed in migrating fibroblasts derived from Cx43 KO mice [105]. Cx43 also inhibits FAK signaling and—perhaps not surprisingly considering the close links between FAK/RhoA/p38-signaling—the inhibition of FAK signaling increases TNT levels. However, this finding does contrast with that of a study showing that FAK inhibition can reduce TNT levels in squamous cell carcinoma cells [106]. The frequent observation of the cell line-dependent regulation of TNTs further cements the idea that TNT formation is a highly complex process involving various cell context-dependent pathways.

A clear observation from the kinase inhibition experiments was that although WT cells had more TNTs under basal conditions, KO cells responded more potently to the drugs and induced higher levels of TNTs than WT cells. The underlying reason is not clear, but one can speculate that under basal conditions, Cx43 promotes the formation of TNTs (to maintain contact and synchrony in the cell population), whereas, upon stress, Cx43 can hinder the formation of new TNTs (to avoid the dissemination of negative signals and circumvent the damage). Indeed, these “kiss of life” and “kiss of death” phenomena are well-described for classic gap junctions mediating the bystander effect between adjacent cells, such as in cancer progression and the therapeutic response [4,107]. The molecular mechanism controlling this aspect remains unclear, but a balance between multiple cell signaling pathways likely controls the overall fate of the cell in terms of TNT establishment.

It remains unclear whether channel-dependent or -independent mechanisms are the dominant Cx43-mediated pathways influencing TNTs. From the literature, multiple hypotheses can be postulated, including a role for the independently synthesized truncated forms of Cx43 in directly regulating the intracellular signaling pathways affecting TNTs. Our study supports this hypothesis. GJA1-20k arranges actin (maintaining F-actin structure) to guide Cx43 delivery [15], as well as potentially that of other proteins involved in membrane dynamics. The Cx43/ZO-1 complex also regulates endothelial F-actin architecture [108]. Additionally, GJA1-20k contains a microtubule-binding motif that promotes microtubule polymerization [11,105,109,110]. Expression of the truncated Cx43 form GJA1-20k did seem to induce TNTs in our KO cells, although additional experiments are needed to dissect the molecular pathway and draw firm conclusions.

Apart from regulating the function of TNTs, GJIC may also regulate their formation. Indeed, the inhibition of GJIC using chemical inhibitors suggested that fewer TNTs were formed, specifically in WT and not KO cells. However, these chemical inhibitors affect a number of other pathways that may influence TNTs, and our preliminary experiments using a more specific peptide-based approach to inhibit Cx43 channels did not prove fruitful. This issue might be deciphered by the use of other connexin species to restore GJIC. Moreover, it should be noted that the formation of TNTs seems highly sensitive to cellular conditions, including time in tissue culture, seeding density, and duration of trypsinization and other factors that affect cell health. This variation can be appreciated in our study, because individual biological replicates have been color-/shape-coded to allow the tracing of trends between replicates and experiments performed on different days. Such variation reduces the statistical significance and reinforces the importance of maintaining the cellular conditions as similar as possible.

We also noted significant variation among cell lines. BT549 and Hs578t are triple-negative breast cancer cells (with a mesenchymal phenotype) [111] that display particularly high levels of TNTs. Other triple-negative cell lines, such as SUM159PT (as shown here) and MDA-MB-231, as well as estrogen receptor-positive MCF-7 cells, also express TNTs, but to a lesser extent [58,59,112,113,114]. In this context, comparative gene expression analysis of both WT and Cx43 KO cells may provide valuable insight into the regulatory pathways important for TNT formation.

Finally, Cx43 affects the secretome [89], and our results clearly show that conditioned medium from Cx43 WT cells was a significantly more potent inducer of TNTs than that from KO cells. This suggests that Cx43 can affect TNTs in more distant cells as well as potentially in other cell types. This will be an exciting avenue to explore in the future. Notably, exosomes have been shown to stimulate TNT formation [115]. The silencing of RASSF1A inactivates the RhoB guanine nucleotide exchange factor GEF-H1, leading to Rab11 accumulation and subsequent exosome release, which in turn contribute to TNT formation [116]. An interaction between Cx43 and Rab11 has been described in endocytosis [117], and vesicles containing Rab11 and Cx43 are transported between mitotic cells and adjacent cells via actin-based plasma membrane bridges [52]. A Rab11a–Rab8a cascade has been shown to regulate TNT formation through vesicle recycling [118]. Cx43 is a functional constituent of exosomes [8,9], and Cx43 phosphorylation may promote exosome release [119]. The link between Cx43, exosomes, and TNTs warrants further investigation.

In conclusion, this work highlights a direct link between Cx43, TNTs, and cell signaling pathways that may play an important role in cancer progression and provides clues as to why Cx43 is often found to be pro-tumorigenic in highly malignant and metastatic cancers [4]. Nonetheless, Cx43 is implicated in many other diseases [2]. Notably, Cx43 plays a central role in ischaemic heart disease, which is a condition recently described to impact TNT-mediated communication among cardiac cells [120]. Thus, elucidation of the Cx43–TNT cell communication axis is an important future endeavor with potential therapeutic opportunities.

## 4. Materials and Methods

### 4.1. Cx43 Knockout and Overexpression of Cx43 and GJA1-20k

#### 4.1.1. Generation of an All-in-One High-Fidelity Cbh-hfCas9-2A-eGFP CRISPR Plasmid

pSpCas9(BB)-2A-GFP (PX458), a gift from Feng Zhang (Addgene plasmid #48138; http://n2t.net/addgene:48138; RRID:Addgene_48138), allows the expression of CRISPR targeting sequences together with an EGFP-linked form of Cas9 to control transfection and FACS selection [66]. To minimize putative off-target cleavage, we replaced Cas9 with a high-fidelity Cas9 gene [67] amplified from the V-T7-humanSpCas9-HF1-NLS-3xFLAG (VP12) vector gifted by Keith Joung (Addgene plasmid #72247; http://n2t.net/addgene:72247; RRID:Addgene_72247). The hSpCas9 gene was removed by digesting the PX458 plasmid with FastDigest AgeI (Thermo Scientific; #ER1461, Waltham, MA, USA) and FseI (New England BioLabs; #R0588S, Hitchin, UK) in CutSmart^®^ Buffer (New England Laboratories; #B7204S): the AgeI cut site is 1238 bp before the FLAG tag, whereas the FseI cut site is 5499 bp after the 2A-EGFP sequence. Primers (with AgeI and FseI restriction sites) were designed to amplify by conventional PCR the SpCas9 hf1 gene and also the T7, SV40 NLS, and FLAG tag sequences (Fw: 5′-GCACCGGTCG GCCGCTAATA CGACTCACTA TAG-3′; Rev: 5′-CTAGGCCGGC CAATCCTGCA GCCTTGTCAT CGTCATC-3′). The reverse primer was designed to remove the stop codon to allow continuous in-frame translation of the self-cleaving EGFP present in the receptor vector. We used Phusion Hot Start High-Fidelity DNA Polymerase (2 U/μL) (New England Laboratories; #M0530L) under the recommended conditions and with the following PCR protocol: an activation step (98 °C for 30 s), followed by 30 cycles of denaturation (98 °C for 10 s), annealing (54 °C for 30 s), and elongation (72 °C for 65 s), with a final elongation step (72 °C for 10 min). PCR amplification products were digested with FseI and AgeI and ligated into the digested PX458 using the T4 DNA Ligase System (Thermo Scientific). The new plasmid, named Cbh-hfCas9-2A-eGFP, was initially screened by KpnI and SalI double digestion, and the final plasmid was sequenced using the T7 promoter primer (5′-TAATACGACT CACTATAGGG-3′) and a reverse EGFP N-terminal primer (5′-CGTCGCCGTC CAGCTCGACC AG-3′).

#### 4.1.2. Cbh-hfCas9-2A-eGFP CRISPR System Targeting Cx43

The gap junction protein alpha 1 (*GJA1*) gene sequence, encoding the Cx43 protein, was imported from the Ensembl Database (ENSG00000152661, GRCh38 (hg38, *Homo sapiens*)). Single small guide RNAs (sgRNAs) targeting the protein-encoding exon 2 region were designed using the publicly available tool Benchling (https://benchling.com), which predicts the 5′-NGG-3′ protospacer adjacent motifs (PAMs) in a given sequence and the potential sgRNAs in both strands, giving their on-target and off-target scores. We choose 20-nucleotide-long sgRNAs with acceptable on-target and off-target scores, ensuring that the guides did not contain a BbsI restriction site. The sequences were as follows: Pair 1, 5′-CACCGAAGCC TACTCAACTG CTGG-3′/5′-AAACTCCAGC AGTTGAGTAG GCTTC-3′; Pair 2, 5′-CACCGACAGC GGTTGAGTCA GCCTG-3′/5′-AAACCAGGCT GACTCAACCG CTGTC-3′.

The sgRNA oligo duplex was generated using 0.5 μL of T4 Polynuclease Kinase (10 U/μL) (Thermo Scientific; #EK0031), 1 μL of 10× T4 Ligase Buffer (Thermo Scientific; #B69), 10 μM of forward sgRNA oligo, 10 μM of reverse sgRNA oligo, and 6.5 μL of nuclease-free water. Phosphorylation and annealing involved an initial step of 30 min at 37 °C, followed by 5 min at 95 °C and a 5 °C/min decrease until 25 °C. For cloning, the Cbh-hfCas9-2A-eGFP vector was digested with BbsI, which generated compatible ends with the BbsI-specific overhangs of the sgRNA oligo duplex. The reaction comprised 100 ng of Cbh-hfCas9-2A-eGFP, 1 μL of BbsI (10 U/μL) (Thermo Scientific; #ER1011), 0.5 μL of T4 Ligase (1 U/μL) (Invitrogen, Thermo Scientific; #15224090), 2 μL of 10× Tango Buffer (Thermo Scientific; #BY5), 0.1 μM of the annealed and phosphorylated sgRNA oligo duplex, 0.5 mM of dithiothreitol (DTT) and ATP, and nuclease-free water to a total volume of 20 μL. Six 10-min cycles (5 min at 37 °C, then 5 min at 21 °C) were followed by a 10-min heat-inactivation step at 65 °C. DH5α *E. coli* cells were transformed with standard procedures, and plasmid isolation was performed using the commercial GeneJET Plasmid Miniprep kit (Thermo Scientific; #K0502). Colonies were screened with SalI + BbsI double digest, and positive clones were verified by sequencing from the U6 promoter (5′-ACTATCATAT GCTTACCGTA AC-3′).

#### 4.1.3. Generation of KO Clones

Cells were transfected with Lipofectamine 3000 (Thermo Scientific; #L3000015) following the manufacturer’s instructions. Seventy-two hours later, the cells were sorted (BD FACSAria II cell sorter (BD Biosciences, San Jose, CA, USA)) and seeded at 1 cell/well in 96-well plates previously prepared with 150 μL of conditioned medium per well (50% normal medium + 50% conditioned medium). Clones were screened for Cx43 expression by western blot and immunofluorescence imaging.

#### 4.1.4. Overexpression of Cx43 and GJA1-20k

Cx43-IRES-GFP in the retroviral pLPCX vector (Clontech, Mountain View, CA, USA) has been previously described [10] and is available as Addgene plasmid #65433. GJA1-20k was cloned from pDEST-EGFP-Cx43-ML-N1 (Addgene plasmid #49860; http://n2t.net/addgene:49860; RRID:Addgene_49860) as a template into pEGFP-N2 (Clontech) plasmid by PCR cloning (FW-XhoI: 5′-GCGCTCGAGA TGCTGGTGGT GTCCTTGGTG TCC-3′; and Rv-HindIII: 5′-GCGAAGCTTC TAGATCTCCA GGTCATCAGG CCG-3′) and digested with XhoI and NotI into the pLPCX vector and verified by sequencing. Transfection, viral collection, and infection were performed as previously described [121]. Cells were sorted by flow cytometry (High Speed Cell Sorter FacsAria (Becton Dickinson, Franklin Lakes, NJ, USA)) to obtain a population entirely comprising EGFP-positive cells.

### 4.2. Cell Culture

Human cancer cells Hs578t, BT-549, and SUM159PT (American Type Culture Collection) were grown in standard Dulbecco’s modified Eagle’s medium (DMEM) (Thermo Scientific) supplemented with 10% (*v*/*v*) heat-inactivated fetal bovine serum (FBS) (Labclinics, Barcelona, Spain) and penicillin (100 U/mL)–streptomycin (100 U/mL) (Thermo Fisher Scientific) in a humidified incubator (37.0 °C, 5% CO_2_). Hs578t cells were additionally supplemented with human insulin (10 µg/mL) (Sigma-Aldrich, Deisenhoffen, Germany) to support growth.

### 4.3. TNT Analysis

Cells (75,000) were seeded into 6-well plates. Unless otherwise stated, the medium was replaced the following day with either normal DMEM medium, low-serum DMEM (1% FBS), or drug-/peptide-supplemented DMEM. Then, after overnight incubation, images were taken with a Nikon Eclipse TE2000-S inverted microscope at 10× magnification. Between 5 and 10 images were obtained for each condition in each independent experiment, and the numbers of cells and TNTs were counted to calculate the ratio of TNTs/cell.

### 4.4. Drug Treatments

Hs578t WT and KO cells (75,000 cells/well) were seeded in a 6-well plate. Drugs were applied and the cells were incubated overnight (16 h) or for 8 h (for carbenoxolone, PF 431396, IPA-3, and latrunculin A). The final concentrations of the drugs were as follows: meclofenamate sodium (Chem Cruz; #sc200532A) 25 µM; forskolin (Sigma-Aldrich; #F6886), 30 µM; latrunculin A (Tocris; #3973, Bristol, UK), 10 nM; SB203580 (Selleckchem; #S1076, Houston, TX, USA), 25 µM; carbenoxolone (Sigma-Aldrich; #C4790), 25 µM; Y-27632 (Selleckchem; #S1049), 10 µM; IPA-3 (Selleckchem; #S7093), 2.5 µM; 8-Br-cAMP (Abcam; #ab141448, Cambridge, UK), 100 µM; H89 dihydrochloride (Sigma-Aldrich; #C4790), 20 µM; and PF 431396 (Tocris; #4278), 5 µM.

### 4.5. Peptide Treatments

All peptides were custom-synthesized by GeneScript to a minimum purity of 85% and shipped as a lyophilized powder. The peptides were resuspended in sterile 1× phosphate-buffered saline (PBS) or dimethyl sulfoxide (DMSO) and added to media at 25 µmol/L. The cells were incubated overnight. The next morning, 10 random photomicrographs were taken, and the TNT/cell ratio was calculated. All peptides were screened twice, with the exception of Tat-Cx43 (amino acids 266–283), for which four independent experiments were performed. The following peptides were tested: TAT-Cx43(266–283), YGRKKRRQRR RAYFNGCSSP TAPLSPMSP; TAT-Cx43(274–291), YGRKKRRQRR RPTAPLSPMS PPGYKLVTG; TAT-Cx43(266–283REV), YGRKKRRQRR RAPSMPSLPA TPSSCGY; ACT1 RQPKIWFPNR RKPWKKRPRP DDLEI; ACT1R, RQPKIWFPNR RKPWKKIELD DPRPR; Peptide5, VDCFLSRPTK T; CT10, SRPRPDEI; Gap27, SRPTEKTIII; and Gap27rev, IIFITKETPS.

### 4.6. Conditioned Medium Treatment

WT or KO Hs578t cells (500,000 cells) were seeded in 100-mm cell culture dishes and cultured for 4 days. Conditioned media were collected and filtered through a 0.2-µm filter to ensure sterility. Three wells of WT cells and three of KO cells were seeded in a 6-well plate at 75,000 cells/well. The cells were allowed to grow for 24 h. Afterward, the media was aspirated, the wells washed with 2 mL of 1× PBS, and the medium of each well replaced with complete DMEM (control), conditioned medium from WT cells (CM-WT), or conditioned medium from KO cells (CM-KO). The cells were incubated overnight before the TNT numbers were quantified.

### 4.7. Protein Extraction and Quantification

Cells were lysed with radioimmunoprecipitation assay (RIPA) Lysis Buffer (50 mM Tris-HCl, pH 7.4, 150 mM NaCl, 1% Triton X-100, 1% sodium deoxycholate, 0.1% SDS, 1 mM ethylenediaminetetraacetic acid (EDTA)) (Santa Cruz Biotechnology, Dallas, TX, USA) supplemented with PhosSTOP and Complete Phosphatase/Protease Inhibitor Cocktails (Roche Diagnostics GmbH, Mannheim, Germany). Protein was quantified using the Pierce BCA Protein Assay Kit (Thermo Fisher Scientific).

### 4.8. Western Blotting

Complete protein extracts (30 µg) were loaded onto SDS-PAGE gels and electrophoretically transferred to polyvinylidene fluoride (PVDF) membranes (Thermo Fisher Scientific). Then, the membranes were blocked with either 5% (*w*/*v*) non-fat dry milk or 5% (*w/v*) bovine serum albumin (BSA) (Sigma-Aldrich) in phosphate-buffered saline buffer containing 0.1% Tween-20 (Sigma-Aldrich) and incubated with primary antibody overnight at 4 °C. horseradish peroxidase (HRP)-conjugated secondary antibody incubation was performed at room temperature for 1 h. Protein immunodetection was performed using ECL™ Western Blotting Detection Reagents (GE Healthcare, Buckinghamshire, UK). The following primary antibodies were used: Cx43 (Sigma-Aldrich; #C6219; 1:8000), vinculin (Sigma-Aldrich; #V4505; 1:1000), ROCK1 (Santa Cruz Biotechnologies,; #sc-17794; 1:100), ezrin (Santa Cruz Biotechnologies; #sc-5875; 1:1000), vimentin (Santa Cruz Biotechnologies; #sc-6260; 1:1000), and RhoA (Cytoskeleton Inc., Denver, CO, USA; #ARH03; 1:1000). The secondary antibodies were rabbit anti-mouse horseradish peroxidase and goat anti-rabbit horseradish peroxidase (Life Technologies, Carlsbad, CA, USA; 1:10,000).

### 4.9. Immunofluorescence Microscopy

Cells were seeded on sterile glass coverslips (20,000 cells/coverslip) and grown to semi-confluency. After 28–48 h, they were washed once with 1× PBS and fixed with 4% formaldehyde for 15 min at room temperature. After two additional washes, the cells were permeabilized and blocked in PBS containing 20% goat serum and 0.5% Triton X-100 for 1 h at room temperature (RT). Afterward, the cells were stained with the ActinRed™ 555 ReadyProbes^®^ Reagent (Thermo Fisher Scientific; #R37112), following the steps given by the manufacturer. From this step onward, we protected the dish from direct light. Then, the cells were incubated for 1 h at room temperature with a rabbit anti-Cx43 antibody (Sigma; #C6219) diluted 1:500 in antibody buffer (1× PBS containing 5% goat serum). Cells were washed three times with 1× PBS and incubated for 1 h at room temperature with secondary antibody (Alexa Fluor^®^ 488 goat anti-rabbit [Sigma-Aldrich] diluted at 1:1000 in antibody buffer). Then, the cells were washed three times with PBS and mounted by placing the coverslips cell-side down on glass slides with a drop of ProLong Diamond Antifade Mountant containing DAPI (Life Technologies; #P36962) to automatically stain the nuclei. Samples were stored at 4 °C in the dark until analysis. Finally, samples were observed using an Olympus FSX100 fluorescent microscope or confocal microscope Olympus FV1000 as indicated.

### 4.10. F-actin/G-actin Ratio Determination

Cells (70,000) were seeded in 24-well culture dishes. The cells were cultured in DMEM containing 10% or 1% FBS overnight for cells undergoing non-stimulated or stimulated conditions, respectively. The next day, the 1% FBS-DMEM was replaced with normal 10% FBS-DMEM for 3 h to stimulate the cells. This was followed by fixation of the cells in 4% paraformaldehyde for 15 min at room temperature, permeabilization in 0.5% Triton X-100 for 5 min, and PBS rinsing. An incubation solution of 2 drops/mL ActinRed 555 (Life Technologies) to stain F-actin and 10 μg/mL Alexa Fluor 488-labeled DNAseI (Life Technologies) to stain monomeric G-actin was added to the cells for 1 h. After three washing steps with PBS, images of the F-/G-actin expression were obtained using an Olympus FSX100 inverted microscope at 5× magnification. The relative ratio of F-/G-actin fluorescence intensity was determined using ImageJ.

### 4.11. Parachute Assay

Cells at 70–90% confluency were prelabeled in DMEM containing 10 µg/mL DiI (C18) (a red fluorescent long-chain dialkylcarbocyanine tracer dye), 5 µg/mL calcein AM (a green fluorescent probe that can pass through gap junctions), and 2.5 mM probenecid or 10 µM verapamil (an anion transport inhibitor that minimizes calcein leakage from cells). After 30-min incubation at 37 °C, the cells were trypsinized and sparsely seeded (1:25 to 1:50) into wells containing 50–70% confluent unlabelled cells. After 4 h of co-culture, all cells were trypsinized, and cell fluorescence was measured by flow cytometry. The transfer of calcein AM from the prelabeled cells to recipient cells via gap junctions can be detected, whereas the red fluorescent DiI remains in the donor cell.

### 4.12. Statistical Analysis

All statistical analyses were performed with GraphPad Prism version 8. Data were tested for normality using the Shapiro–Wilk test. Comparisons of two groups of parametric data were performed with Student’s *t*-test, whereas multiple groups were analyzed by one-way ANOVA using Tukey’s post-hoc test. Statistical significance was set at *p* < 0.05. All data are displayed as mean ± SEM.

## 5. Conclusions

Previous work demonstrated the presence of Cx43 in TNTs and that it potentially regulated the passage of small molecules between cells through gap junctions. However, TNTs are generally thought to transfer much larger proteins and organelles, which would not be compatible with the small size limit of gap junctions of around 1.2 kDa. This work identified Cx43 as a direct regulator of TNT formation. We also established several key pathways linked to Cx43, including p38, PKA signaling, ROCK signaling, and FAK signaling, which regulate TNTs in a Cx43-dependent manner. Elucidation of the role of Cx43 in TNTs brings conceptual advances linking Cx43 to pro-tumorigenicity. A better understanding of TNT formation could provide a novel avenue for cancer targeting.

## Supplementary Materials

The following are available online at https://www.mdpi.com/2072-6694/12/10/2798/s1, Figure S1: GJIC in BT549 cells, Figure S2: F/G actin ratio and changes in protein expression, Figure S3: Peptide Screen, Figure S4: GJIC in Hs578t cells, Figure S5: original western blot figures. Video S1: 24-h time-lapse video of BT549 cells.
