# Supplementary material for: Cx43 and Associated Cell Signaling Pathways Regulate Tunneling Nanotubes in Breast Cancer Cells
Source: Cancers (Basel). 2020 Sep 29;12(10):2798. doi: 10.3390/cancers12102798 (PMC7601615; doi:10.3390/cancers12102798)
Supplement: Supplementary file 1 [file cancers-12-02798-s001.zip › Supplementary files Aasen Proof 28sept2020/Supplementary Figures 1_5 Aasen proof.pdf]

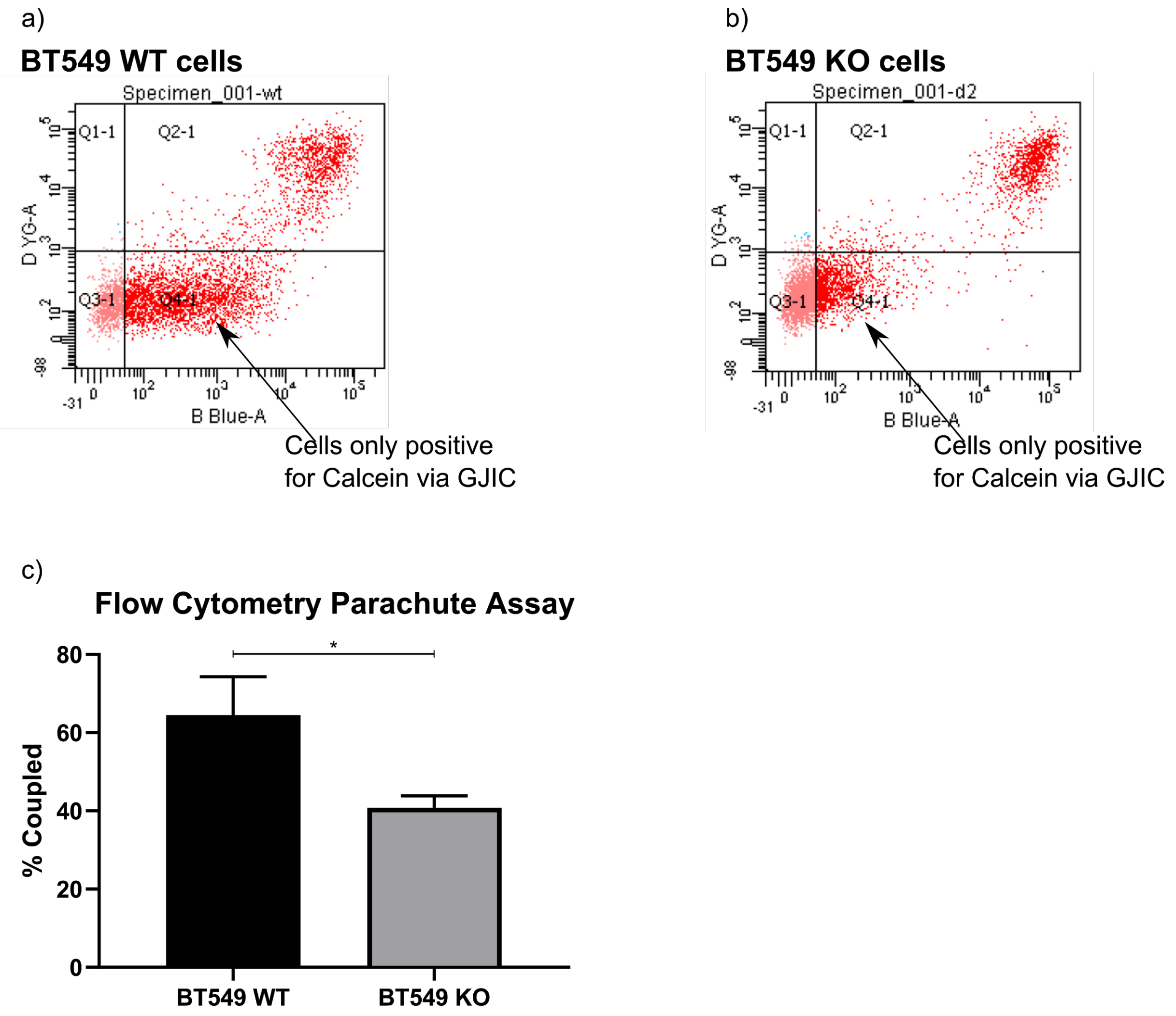

Supplementary Figure S1. GJIC in BT549 cells. Using the parachute assay (preloading with Calcein AM and DiD-red) cells were seeded on non-stained WT or KO cells and analysed by flow cytometry after 5 hours). Example of flow cytometry analysis of WT (**a**) and KO cells (**b**) and a summary of the analysis (n=2) in (**c**). Error bars denote the mean with s.e.m. Significance was tested using one-way t-test to identify if there is a significant reduction of GJIC in KO cells (*$p<0.05$).

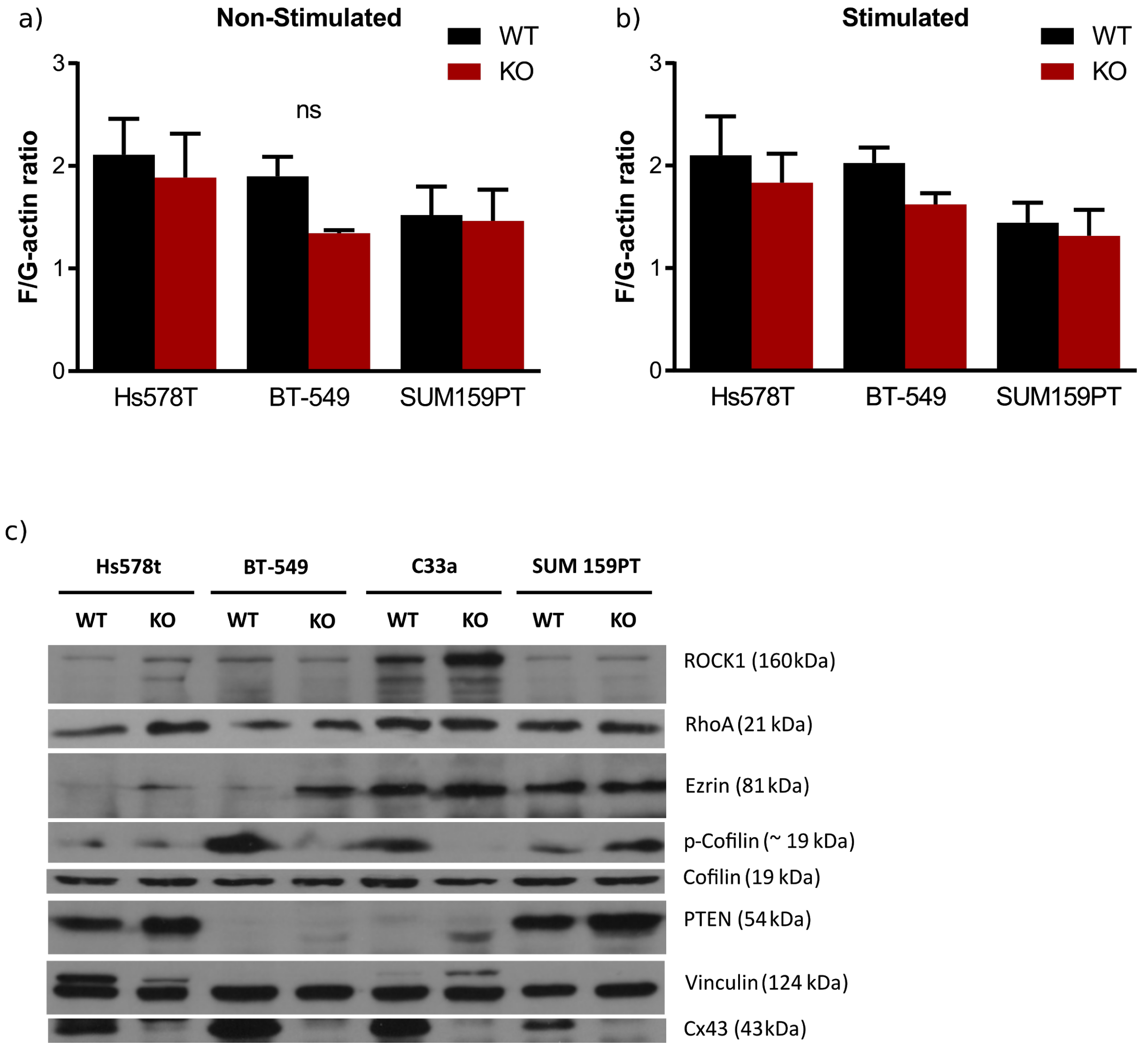

Supplementary Figure S2. F/G actin ratio and changes in protein expression. Analysis of the F/G-actin ratio in Hs578T, BT549, C33a and SUM159PT WT and KO cells exposed to either normal (**a**) or serum-stimulated (**b**) conditions. The F-/G-actin ratio was slightly reduced in Cx43 KO cells compared to WT cells but no significant differences were reached. Data are shown as mean ± SEM of three independent experiments. Student's t-test was performed to calculate statistical significance (ns = not significant). Western blot of Hs578T, BT549, C33a and SUM159PT WT and KO cells (**c**) with Antibody and protein size indicated.

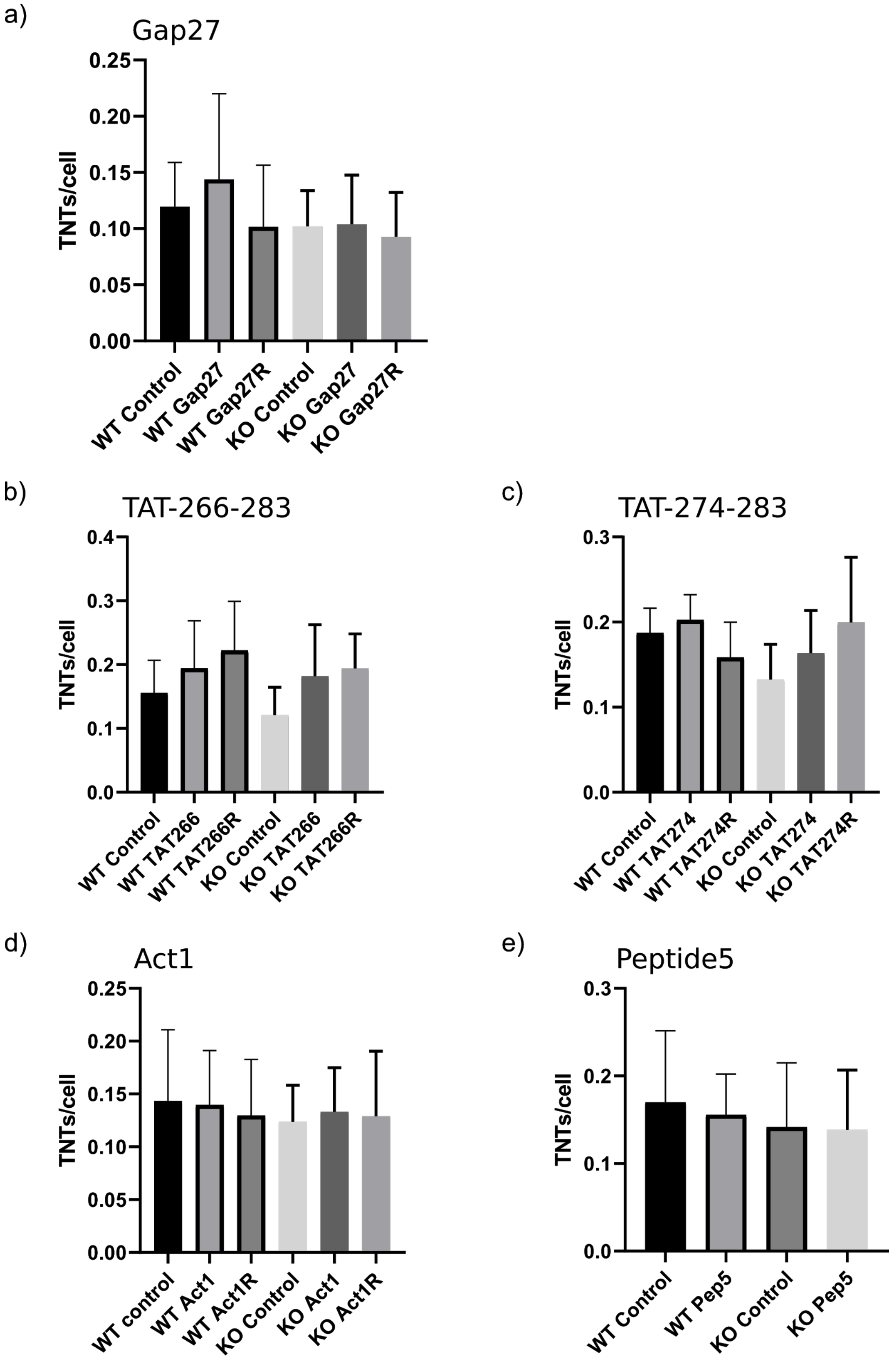

Supplementary Figure S3. Peptide Screen. A preliminary screen of the effect on TNT levels in Hs578t cells upon overnight treatment with 100uM peptides. No clear induction or reduction by the specific peptide and not the reverse (R) control peptide was seen. Error bars represent Standard Deviance (SD) of the analysis of n=5-10 images.

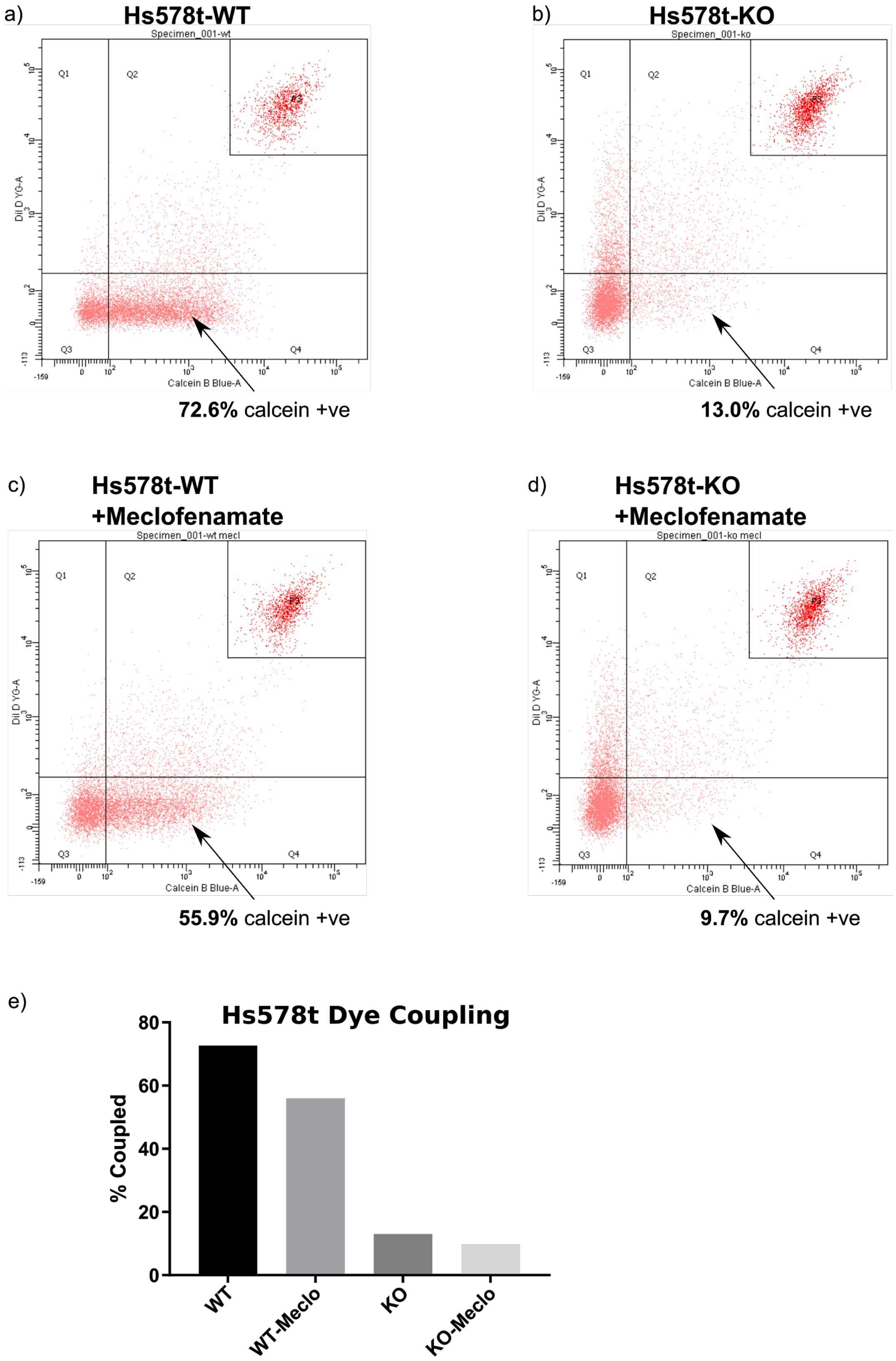

Supplementary Figure S4. GJIC in Hs578t cells. Using the parachute assay (preloading with Calcein AM and DiD-red) cells were seeded on non-stained WT or KO cells and analysed by flow cytometry after 5 hours). Example of flow cytometry analysis of WT (**a**), KO cells (**b**), WT+Meclofenamate (**c**), KO cells + Meclofenamate (**d**) and a graphical summary of the analysis (**e**).

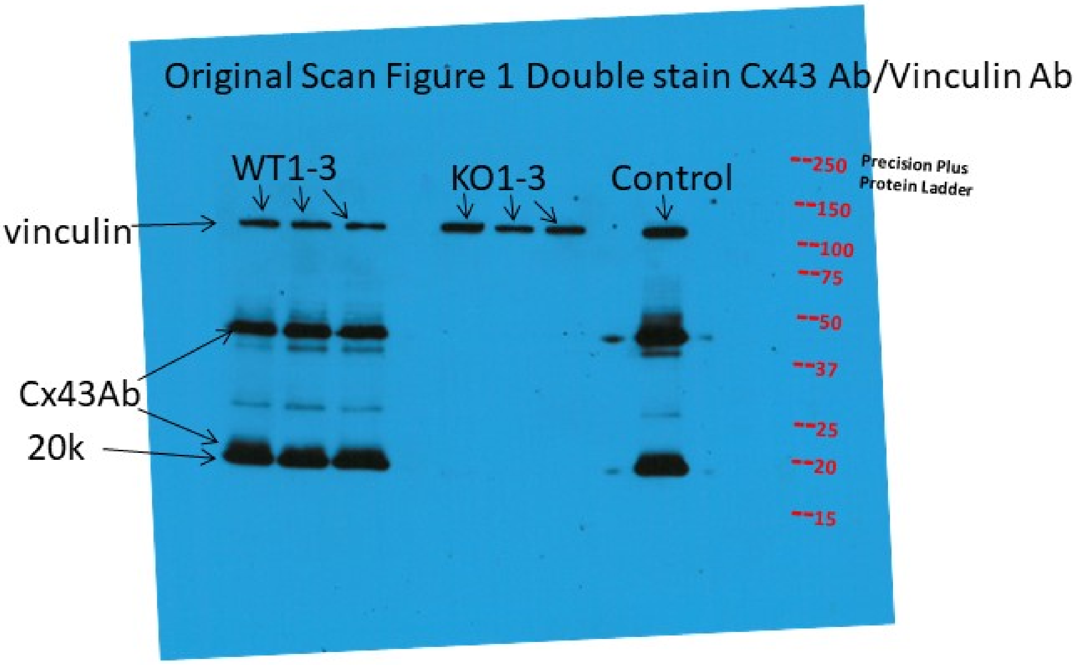

Original Scan Figure 1 Double stain Cx43 Ab/Vinculin Ab

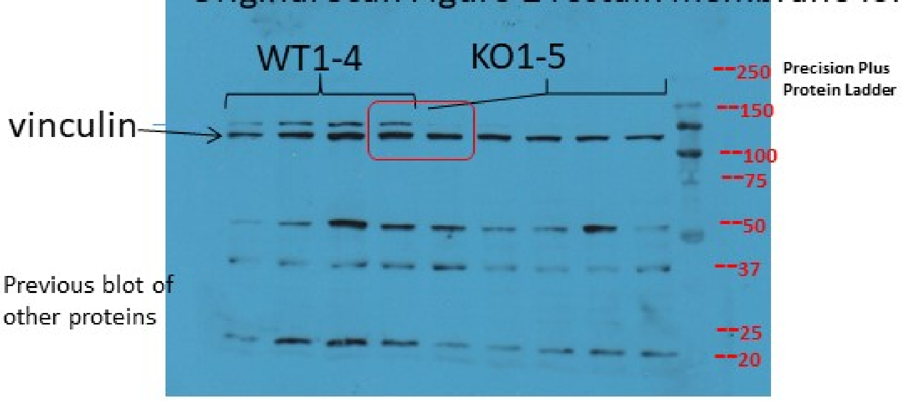

Original Scan Figure 2 restain membrane for Vinculin

Figure 2 restain membrane for Cx43

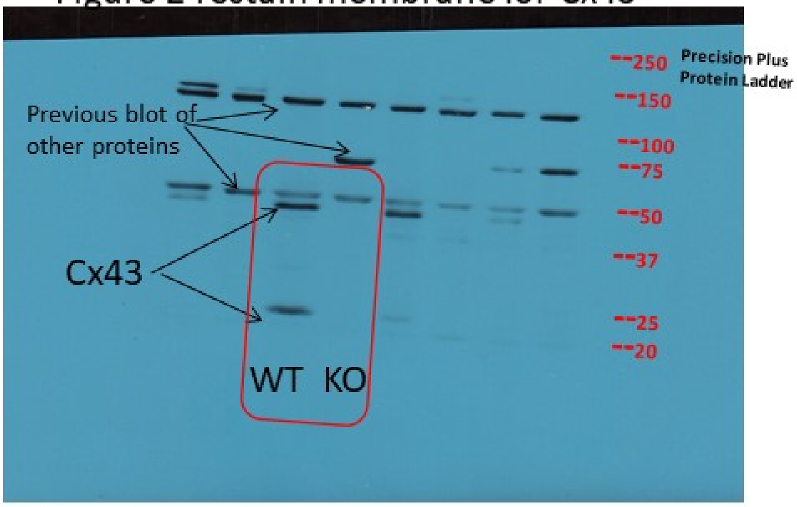

Original Scan Figure 3 membrane cut in 2 independent pieces: Cx43

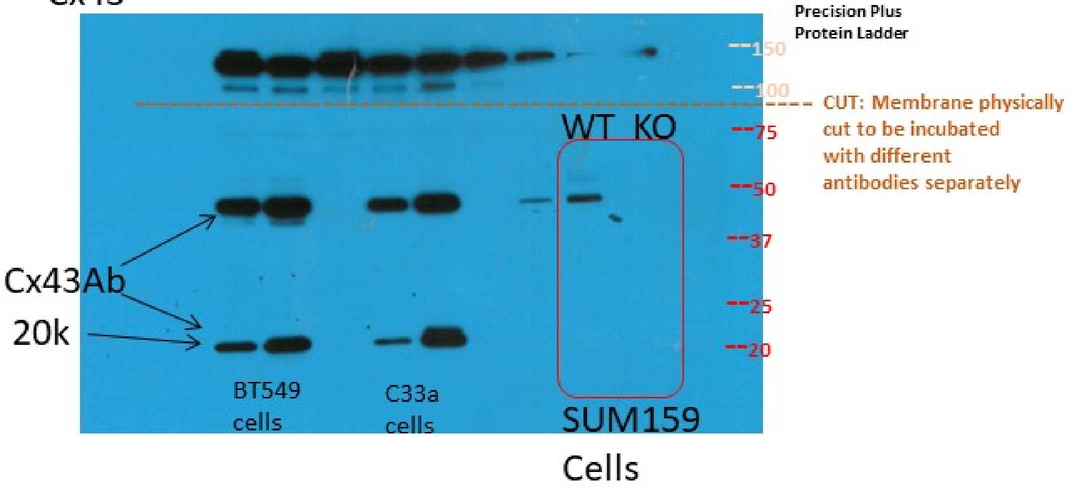

Original Scan Figure 3 membrane cut in 2 independent pieces : Vinculin

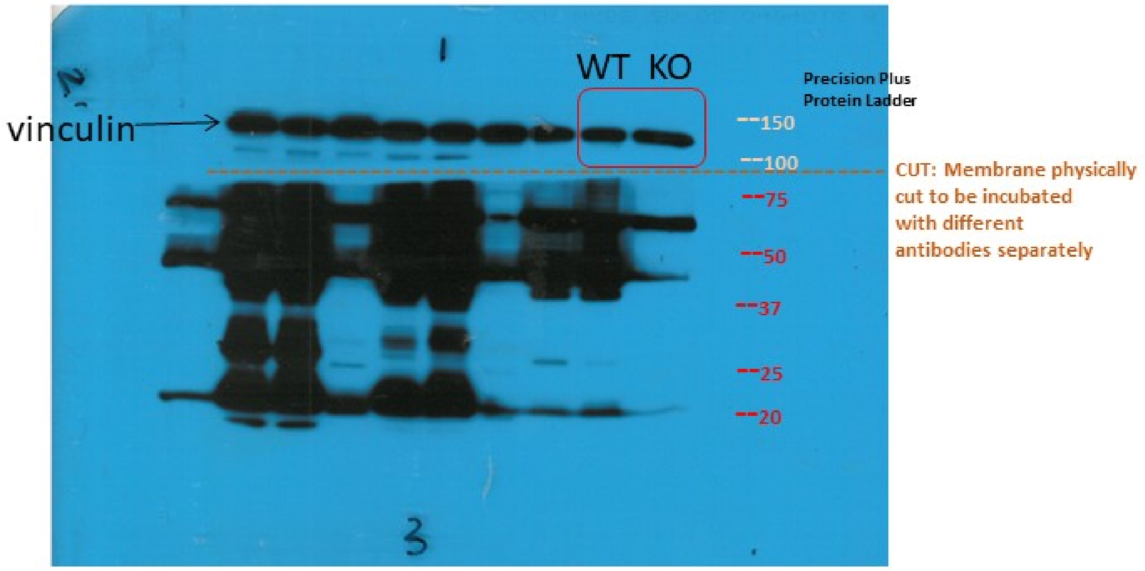

Supplementary Figure S5. Original western blot figures. Note that some blots have been re-stained with other antibodies and therefore display additional bands.
